# Association of endometriosis with asthma: a study of the NHANES database in 1999–2006

**DOI:** 10.1186/s41043-024-00541-3

**Published:** 2024-04-09

**Authors:** Guangxin Pan, Pei Zhang, Sha Li, Lanlan Cao, Changqun Yang

**Affiliations:** 1 Department of Obstetrics and Gynecology, Tongji Medical College, The Central Hospital of Wuhan, Huazhong University of Science and Technology, No 26. Shengli Street, Jiang’an District, Wuhan, 430014 Hubei Province P. R. China; 2 Key Laboratory for Molecular Diagnosis of Hubei Province, Tongji Medical College, The Central Hospital of Wuhan, Huazhong University of Science and Technology, Wuhan, 430014 P. R. China

**Keywords:** Endometriosis, Asthma, NHANES database, Cross-sectional study, Adult women

## Abstract

**Objective:**

Asthma is a chronic inflammatory disease of the airways with a gender differences in the prevalence after puberty. Recent studies have reported a relationship between asthma and endometriosis, possibly related to the immune response mechanisms, but the evidences are limited and inconsistent. Herein, this research aimed to investigate the association of endometriosis with asthma based on the representative population in the United States (U.S.) to provide some reference for further exploration on mechanism of gender difference in asthma.

**Methods:**

In this cross-sectional study, data of women aged ≥ 20 years old were extracted from the National Health and Nutrition Examination Survey (NHANES) database in 1999–2006. Weighted univariate and multivariate logistic regression analyses were utilized to explore the association of endometriosis with asthma. The multivariate models adjusted for covariates including age, race, education level, marital status, poverty income ratio (PIR), body mass index (BMI), waist circumference, smoking, estrogen and progesterone hormones use, uterine fibroids, at least one ovary removed, and birth control pills intake. The evaluation indexes were odds ratios (ORs) and 95% confidence intervals (CIs). Subgroup analyses of age, race, BMI, and pregnancy history were also performed.

**Results:**

Among 5,556 eligible women, 782 had asthma, and 380 had endometriosis. The average age of participants was 37.19 years old, and more than half of them were non-Hispanic White (68.44%). After adjusting for covariates, endometriosis was associated with higher odds of asthma compared with non-endometriosis [OR = 1.48, 95%CI: (1.10–1.99)]. This relationship was also found in 40–49 years old [OR = 2.26, 95%CI: (1.21–4.23)], BMI of 25-29.9 kg/m^2^ [OR = 2.87, 95%CI: (1.52–5.44)], and pregnancy history [OR = 1.44, 95%CI: (1.01–2.06)] subgroups.

**Conclusion:**

Endometriosis had a positive association with asthma in adult women. Females aged 40–49 years old, with BMI of 25-29.9 kg/m^2^ and had a history of pregnancy should take care about monitoring endometriosis to reduce the potential risk of asthma. Further studies are still needed to clarify the causal association between endometriosis and asthma.

**Supplementary Information:**

The online version contains supplementary material available at 10.1186/s41043-024-00541-3.

## Introduction

As a chronic inflammatory disease of the airways, asthma can bring about wheezing, chest tightness, shortness of breath, and coughing [1]. Epidemiological data on the prevalence, incidence, and severity of asthma appear to suggest gender differences in the risk of developing asthma [2]. Basing on the United States (U.S.) Centers for Disease Control and Prevention (CDC), 25 million persons suffer from asthma, in which 12 million were female adults and 7.3 million were male adults [3]. Among European countries, Japan, and the U.S., the prevalence of asthma after puberty in females is higher than that in males [4, 5]. Although sex hormones ratio in women to those in men may play an important role in etiology, pathogenesis, and clinical manifestations of asthma, evidences connecting sex hormones with asthma remain equivocal [6–8].

Endometriosis affects about 190 million females especially at reproductive age worldwide, which is an inflammatory and chronic gynecologic disease, and is characterized by endometrial-like tissue presenting outside the uterus [9, 10]. Patients with endometriosis seem to have higher risks of multisite pain, infertility, as well as other comorbidities [11]. Recent studies have reported the comorbidity of asthma and endometriosis, but the corresponding evidences are limited and inconsistent [12, 13]. Sinaii et al. [14] found that comparing with the published occurrence rates of allergies and asthma in general female populations in the U.S., they are higher in female adults with endometriosis, about 12%. Peng et al. [15] suggested that women of reproductive age who have asthma are at higher risk of developing endometriosis comparing with those who do not have asthma. Differently, Ferrero et al. [6] conducted a case-control study, showing a similar prevalence of asthma in females with and without endometriosis. Additionally, the biological mechanisms underpinning this relationship remains elusive. Up to now, the most widely accepted theory is that development of endometrial lesion is related to the dysfunction of the immune system, which affects the expression of particular cytokines [16]. The immune responses involving T helper (Th) 1/Th2 and Th17/regulatory T cells (Treg) have been reported to be associated with endometriosis [17, 18]. Besides, Th2 cells overproducing Th cytokines plays a key role in asthma’s pathophysiology [19].

Given the mechanistic link between endometriosis and asthma, as well as the inconsistency of existing epidemiological studies, this research aimed to discuss the association of endometriosis with asthma based on the representative population in the U.S. We hope our findings may provide some reference for further exploration of mechanism of the gender difference in asthma.

## Methods

### Study population

In this cross-sectional study, data of women were extracted from the National Health and Nutrition Examination Survey (NHANES) database in 1999–2006. The NHANES is conducted by the National Center for Health Statistics (NCHS) and the Centers for Disease Control and Prevention (CDC) jointly with the aim of assessing nutritional and health status of noninstitutionalized population in the U.S. The database includes a complex, multistage and stratified probability sample on the basis of selected counties, blocks, households, and persons within households. Information was collected through interviews conducted by the NCHS well-trained professionals in participants’ homes, and extensive physical examinations performed at mobile exam centers (MECs). Details were shown elsewhere https://www.cdc.gov/nchs/nhanes/index.htm.

Initially, 41,474 adult women (aged ≥ 20 years old) in the database were included. After excluding those without information on endometriosis (*n* = 35,917) or asthma (*n* = 1), 5,556 adult women were eligible. The NHANES is approved by the Institutional Review Board (IRB) of NCHS. Since the data are de-identified and publicly available, ethical approval has been waived by the IRB of The Central Hospital of Wuhan.

### Assessment of endometriosis and asthma

According to the NHANES, assessments of endometriosis and asthma were using the Reproductive Health (RHQ_D) and the Medical Conditions (MCQ), respectively. Endometriosis was identified by a positive answer to the question “Has a doctor or other health professional ever told you that you had endometriosis? (Endometriosis is a disease in which the tissue that forms the lining of the uterus/womb attaches to other places, such as the ovaries, fallopian tubes, etc.)” [20] Asthma is defined by the positive answers to the question “Has a doctor or other health professional ever told you that you have asthma?” [21].

### Data collection

In the NHANES, demographic variables, including age, race, education level, marital status, and family poverty income ratio (PIR), were collected through questionnaires. Body mass index (BMI) (kg/m^2^) of all candidates were recorded at the mobile examination center (MEC) by a trained examiner. The variable of cigarette smoking, and other tobacco use was defined by smoking ≥ 100 cigarettes in their entire life. Data on whether individuals had sexual intercourse was also included. In addition, several clinical data were also collected through questionnaires in the NHANES. Using estrogen and progesterone hormones was that participants ever used or was using these female hormones (any forms of estrogen and progesterone hormones, for example, pills, patch, cream, and injectables, but except birth control methods or use for infertility). Participants were asked if they had uterine fibroids by the following question “Has a doctor or other health professional ever told you that you had uterine fibroids? (Yes or no)”. Uterine fibroids are benign (not cancerous) tumors growing in various locations on or within the uterus/womb. Variables of pregnancy history, ovary remove, and birth control pills were assessed by the question “have you ever been pregnant, had at least one of your ovaries removed, and ever taken birth control pills for any reason? (Yes or no)”. Information on “whether the participant is currently pregnant” was also collected.

### Statistical analysis

Continuous variables were expressed as mean ± standard error (S.E), and weighted t test was employed for the comparation between two groups. Categorical variables were expressed by frequency and constituent ratio [n (%)], and weighted chi-square test (𝜒^2^) was used for comparation. All NHANES analyses were weighted, as recommended, to represent the U.S. population [22]. In brief, the NHANES full sample 4 years MEC exam weight (WTMEC4YR) and WTMEC2YR were used for analyses in the current study. The calculation of sample weight for combining data in 1999–2002 was 1/2 × WTMEC4YR, and that for combining data in 2003–2006 was 1/2 × WTMEC2YR.

Weighted univariate logistic regression was utilized to screen potential confounders. Then we used weighted univariate and multivariate logistic regression analyses to investigate the association of endometriosis with asthma. Model 1 only adjusted for age. Model 2 adjusted for demographic variables, including age, race, education level, marital status, PIR, BMI, and waist circumference. Model 3 adjusted for all the covariates selected through weighted univariate logistic regression analysis (*P* < 0.05), including age, race, education level, marital status, PIR, BMI, waist circumference, cigarettes smoking, estrogen and progesterone hormones use, uterine fibroids, at least one ovary removed, and birth control pills use [20, 23, 24]. Subgroup analyses of age, race, BMI, and pregnancy history were also performed to assess the above relationship. The standards of classification for age subgroup and race subgroup were according to menopause or not, and the NHANES criteria, respectively.

The evaluation indexes were odds ratios (ORs) and 95% confidence intervals (CIs). Statistical significance was recognized when *P* < 0.05. Analyses were conducted using SAS v. 9.4 (SAS Institute, Cary, North Carolina). Variables with missing data were shown in Supplementary Tables 1, and were interpolated using multiple imputation method.

## Results

### Characteristics of adult women

Among 5,556 eligible women, 782 had asthma. The characteristics of eligible women were shown in the Table 1. The mean age of total participants was 37.19 years old, in which 1,869 (26.89%) participants were under 30 years old, 1,560 (28.78%) between 30 and 39 years old, 1,463 (30.53%) between 40 and 49 years old, and 664 (13.80%) older than 50. The majority of females were non-Hispanic White [2,567 (68.44%)], followed by Mexican American [1,314 (7.91%)], other Hispanic [1,151 (12.28%)], non-Hispanic Black [271 (5.87%)], and other races [253 (5.50%)]. The mean BMI was 28.19 kg/m^2^. A total of 380 (9.02%) females had endometriosis, whereas 5,176 (90.98%) did not. In addition, PIR, weight, waist circumference, cigarettes smoking, estrogen and progesterone hormones use, uterine fibroids, ovary removed, and birth control pills use were also significantly different between the asthma group and non-asthma group (all *P* < 0.05).

**Table 1:** Characteristics of the study population in the groups with and without asthma

Variables	Total (*n* = 5556)	Asthma		*P*
		Yes (*n* = 782)	No (*n* = 4774)	
Age, years, Mean (S.E)	37.19 (0.18)	36.94 (0.40)	37.24 (0.19)	0.489
Age, years, n (%)^1^				0.188
< 30	1,869 (26.89)	295 (29.50)	1,574 (26.42)	
30–39	1,560 (28.78)	193 (25.32)	1,367 (29.41)	
40–49	1,463 (30.53)	202 (32.14)	1,261 (30.23)	
≥ 50	664 (13.80)	92 (13.03)	572 (13.94)	
Race, n (%)^1^				0.002
Mexican American	1,314 (7.91)	90 (3.63)	1,224 (8.69)	
Non-Hispanic Black	271 (5.87)	46 (6.16)	225 (5.82)	
Non-Hispanic White	2,567 (68.44)	428 (72.76)	2,139 (67.66)	
Other Hispanic	1,151 (12.28)	184 (12.57)	967 (12.23)	
Other races	253 (5.50)	34 (4.89)	219 (5.61)	
Education level, n (%)^1^				0.074
<12th grade	2,966 (60.97)	469 (65.05)	2,497 (60.23)	
High school grade/GED or equivalent	1,244 (23.47)	146 (20.05)	1,098 (24.09)	
College graduate or above	1,346 (15.56)	167 (14.90)	1,179 (15.68)	
Marital status, n (%)^1^				0.053
Married	1,633 (27.72)	269 (31.09)	1,364 (27.11)	
Widowed	84 (1.40)	11 (1.18)	73 (1.44)	
Divorced/separated	3,080 (56.66)	371 (51.19)	2,709 (57.66)	
Never married	759 (14.22)	131 (16.54)	628 (13.80)	
PIR, Mean (S.E)	2.95 (0.04)	2.79 (0.09)	2.98 (0.04)	0.022
Height, cm, Mean (S.E)	163.15 (0.12)	163.33 (0.25)	163.11 (0.14)	0.441
Weight, kg, Mean (S.E)	75.10 (0.47)	78.97 (0.98)	74.40 (0.45)	< 0.001
BMI, kg/m^2^, Mean (S.E)	28.19 (0.17)	29.54 (0.35)	27.95 (0.16)	< 0.001
BMI, kg/m^2^, n (%)^1^				0.002
< 18.5	129 (3.04)	19 (3.25)	110 (3.00)	
18.5–24.9	1,795 (37.68)	219 (32.92)	1,576 (38.54)	
25-29.9	1,580 (25.84)	192 (22.17)	1,388 (26.51)	
≥30	2,052 (33.45)	352 (41.66)	1,700 (31.95)	
Waist circumference, Mean (S.E)	92.30 (0.40)	95.37 (0.84)	91.74 (0.38)	< 0.001
Cigarettes Smoking, n (%)^1^				0.001
Yes	2,110 (42.74)	356 (49.57)	1,754 (41.50)	
No	3,446 (57.26)	426 (50.43)	3,020 (58.50)	
Sexual intercourse, n (%)^1^				0.282
Yes	5,290 (95.77)	754 (96.55)	4,536 (95.63)	
No	266 (4.23)	28 (3.45)	238 (4.37)	
Estrogen and progesterone hormones use, n (%)^1^				0.011
Yes	672 (15.44)	126 (19.64)	546 (14.68)	
No	4,884 (84.56)	656 (80.36)	4,228 (85.32)	
Uterine fibroids, n (%)^1^				0.010
Yes	699 (13.70)	124 (17.46)	575 (13.02)	
No	4,857 (86.30)	658 (82.54)	4,199 (86.98)	
Pregnancy history, n (%)^1^				0.601
Yes	4,695 (79.57)	653 (78.74)	4,042 (79.72)	
No	861 (20.43)	129 (21.26)	732 (20.28)	
At least one ovary removed, n (%)^1^				0.012
Yes	437 (9.71)	78 (13.25)	359 (9.07)	
No	5,119 (90.29)	704 (86.75)	4,415 (90.93)	
Birth control pills, n (%)^1^				0.001
Yes	4,189 (79.75)	643 (85.34)	3,546 (78.74)	
No	1,367 (20.25)	139 (14.66)	1,228 (21.26)	
Current pregnant, n (%)^1^				0.523
Yes	1,182 (8.34)	172 (8.93)	1,010 (8.24)	
No	4,374 (91.66)	610 (91.07)	3,764 (91.76)	
Endometriosis, n (%)^1^				< 0.001
Yes	380 (9.02)	88 (13.50)	292 (8.20)	
No	5,176 (90.98)	694 (86.50)	4,482 (91.80)	

Statistical analysis: t test and χ^2^ test

^1^. weighted %

S.E: standard error, GED: general equivalent diploma, PIR: poverty income ratio, BMI: body mass index

### Association of endometriosis with asthma

We first screened the covariates associated with asthma (Table 2). The results showed that race, education level, marital status, PIR, BMI, waist circumference, cigarettes smoking, estrogen and progesterone hormones use, uterine fibroids, ovary removed, and birth control pills were respectively associated with asthma in adult women (all *P* < 0.05).

**Table 2:** Screening of confounding variables for endometriosis and asthma

Variables	Sample size outcome/total		OR (95% CI)	*P*
Age	782/5556		1.00 (0.99–1.01)	0.490
Race				
Mexican American	90/1314		Ref	
Non-Hispanic White	428/2567		2.57 (1.83–3.61)	< 0.001
Non-Hispanic Black	46/271		2.53 (1.43–4.49)	0.002
Other Hispanic	184/1151		2.46 (1.68–3.60)	< 0.001
Other Race	34/253		2.08 (1.22–3.54)	0.008
Education level				
≤ 12th grade	469/2966		Ref	
College graduate or above	167/1346		0.88 (0.70–1.11)	0.270
High school grade/GED or equivalent	146/1244		0.77 (0.60–0.98)	0.038
Marital status				
Married	269/1633		Ref	
Divorced/separated	371/3080		0.77 (0.64–0.94)	0.012
Widowed	11/84		0.71 (0.31–1.66)	0.429
Never married	131/759		1.04 (0.78–1.39)	0.760
PIR	782/5556		0.93 (0.88–0.99)	0.021
BMI	782/5556		1.03 (1.02–1.04)	< 0.001
Waist circumference	782/5556		1.01 (1.01–1.02)	< 0.001
Cigarettes smoking				
No	426/3446		Ref	
Yes	356/2110		1.39 (1.16–1.66)	0.001
Sexual intercourse				
No	28/266		Ref	
Yes	754/5290		1.28 (0.81–2.01)	0.283
Estrogen and progesterone hormones use				
No	656/4884		Ref	
Yes	126/672		1.42 (1.09–1.86)	0.011
Uterine fibroids				
No	658/4857		Ref	
Yes	124/699		1.41 (1.09–1.83)	0.010
Pregnant history				
No	129/861		Ref	
Yes	653/4695		0.94 (0.75–1.18)	0.602
At least one ovary removed				
No	704/5119		Ref	
Yes	78/437		1.53 (1.10–2.14)	0.013
Birth control pills use				
No	139/1367		Ref	
Yes	643/4189		1.57 (1.22–2.02)	0.001
Current pregnant				
No	610/4374		Ref	
Yes	172/1182		1.09 (0.83–1.44)	0.523

Ref: reference; OR: odds ratio; CI: confidence interval; GED: general equivalent diploma; PIR: poverty income ratio; BMI: body mass index

After adjusting for the selected covariates as well as age, women who had endometriosis seemed to have higher odds of asthma compared to those who without endometriosis [OR = 1.48, 95%CI: (1.10–1.99)]. Similarly, in the analysis of data before multiple imputation, this positive association between endometriosis and asthma was still significant [OR = 1.54, 95%CI: (1.13–2.10)], indicating this result was relatively reliable (Table 3).

**Table 3:** The correlation between endometriosis and asthma

Endometriosis	Model 1			Model 2			Model 3	
	OR (95% CI)	*P*		OR (95% CI)	*P*		OR (95% CI)	*P*
After multiple imputation								
No	Ref			Ref			Ref	
Yes	1.78 (1.36–2.33)	< 0.001		1.77 (1.35–2.32)	< 0.001		1.48 (1.10–1.99)	0.010
Before multiple imputation								
No	Ref			Ref			Ref	
Yes	1.78 (1.36–2.33)	< 0.001		1.80 (1.35–2.41)	< 0.001		1.54 (1.13–2.10)	0.007

OR: odds ratio, CI: confidence interval, Ref: reference

Model 1: crude model;

Model 2: adjusted for age, race, education level, marital status, PIR, BMI, and waist circumference;

Model 3: adjusted for age, race, education level, marital status, PIR, BMI, waist circumference, cigarettes smoking, estrogen and progesterone hormones use, uterine fibroids, at least one ovary removed, and birth control pills use

**Table 4:** The correlation between endometriosis and asthma in age, BMI, pregnancy history, and race subgroups

Subgroups	Sample size outcome/total	Adjusted model	
		OR (95% CI)	*P*
Age < 30	*N* = 295/1869		
No	*N* = 282/1817	Ref	
Yes	*N* = 13/52	0.91 (0.39–2.13)	0.820
Age = 30–39	*N* = 193/1560		
No	*N* = 170/1440	Ref	
Yes	*N* = 23/120	1.64 (0.90–3.02)	0.106
Age = 40–49	*N* = 202/1463		
No	*N* = 161/1320	Ref	
Yes	*N* = 41/143	2.26 (1.21–4.23)	0.012
Age ≥ 50	*N* = 92/664		
No	*N* = 81/599	Ref	
Yes	*N* = 11/65	1.00 (0.47–2.13)	0.992
Mexican American	*N* = 90/1314		
No	*N* = 86/1284	Ref	
Yes	*N* = 4/30	1.28 (0.33–4.98)	0.716
Non-Hispanic Black	*N* = 46/271		
No	*N* = 43/264	Ref	
Yes	*N* = 3/7	4.00 (0.35–45.53)	0.226
Non-Hispanic White	*N* = 428/2567		
No	*N* = 365/2306	Ref	
Yes	*N* = 63/261	1.37 (0.96–1.97)	0.085
Other Hispanic	*N* = 184/1151		
No	*N* = 167/1084	Ref	
Yes	*N* = 17/67	2.18 (0.96–4.94)	0.060
Other Race	*N* = 34/253		
No	*N* = 33/238	Ref	
Yes	*N* = 1/15	0.39 (0.03–5.66)	0.468
BMI < 18.5	*N* = 19/129		
No	*N* = 18/121	Ref	
Yes	*N* = 1/8	0.44 (0.00-Inf)	0.695
BMI = 18.5–24.9	*N* = 219/1795		
No	*N* = 197/1669	Ref	
Yes	*N* = 22/126	1.24 (0.66–2.35)	0.493
BMI = 25-29.9	*N* = 192/1580		
No	*N* = 165/1474	Ref	
Yes	*N* = 27/106	2.87 (1.52–5.44)	0.002
BMI ≥ 30	*N* = 352/2052		
No	*N* = 314/1912	Ref	
Yes	*N* = 38/140	1.28 (0.82–1.99)	0.265
Pregnancy history	*N* = 129/861		
No	*N* = 115/803	Ref	
Yes	*N* = 14/58	1.58 (0.90–2.77)	0.109
Non-pregnancy history	*N* = 653/4695		
No	*N* = 579/4373	Ref	
Yes	*N* = 74/322	1.44 (1.01–2.06)	0.044

BMI: body mass index, OR: odds ratio, CI: confidence interval

Age subgroups: adjusted for race, education level, marital status, PIR, BMI, waist circumference, cigarettes smoking, estrogen and progesterone hormones use, uterine fibroids, at least one ovary removed, and birth control pills use;

Race subgroups: age, education level, marital status, PIR, BMI, waist circumference, cigarettes smoking, estrogen and progesterone hormones use, uterine fibroids, at least one ovary removed, and birth control pills use;

BMI subgroups: age, race, education level, marital status, PIR, waist circumference, cigarettes smoking, estrogen and progesterone hormones use, uterine fibroids, at least one ovary removed, and birth control pills use;

Pregnancy history subgroups: age, race, education level, marital status, PIR, BMI, waist circumference, cigarettes smoking, estrogen and progesterone hormones use, uterine fibroids, at least one ovary removed, and birth control pills use

### Association between endometriosis and asthma in age, race, BMI, and pregnancy history subgroups

The association between endometriosis and asthma was further assessed in different subgroups. As shown in the Table 4, endometriosis was also linked to higher odds of asthma in women aged 40–49 years old [OR = 2.26, 95%CI: (1.21–4.23)], with BMI of 25-29.9 kg/m^2^ [OR = 2.87, 95%CI: (1.52–5.44)], or had pregnancy history [OR = 1.44, 95%CI: (1.01–2.06)].

## Discussion

In the current research, we explored the relationship of endometriosis with asthma. The study results showed that women with endometriosis seemed to have higher odds of asthma. According to the subgroup analyses, the positive association between endometriosis and asthma was also observed in 40–49 years old, BMI of 25-29.9 kg/m^2^, and having pregnancy history subgroups.

At present, evidences on the relationship between endometriosis and asthma in women are limited and inconsistent. Tempest et al. [25] retrospectively collected information on females aged 16–30 years from the Liverpool Women’s Hospital, finding that women with endometriosis who underwent laparoscopy were observed to have higher odds of asthma than women without endometriosis. Similarly, in the present study, we found a positive association between endometriosis and asthma in a representative population of the U.S., after adjusting for relevant covariates. A previous study based on the U.S. population showed asthma in women with endometriosis was more common than that in the general U.S. population [14], but these findings were from a control group of the general population without adjustment for potential confounders [6]. Differently, according to the study conducted by Ferrero et al. [6], a similar prevalence of asthma was observed in women who underwent surgery for benign gynecological disorders between endometriosis and non-endometriosis groups. We speculated that a possible explanation for this difference may be that Ferrero’s findings are limited to a study population only including females who have undergone gynecologic surgery, so that these findings may not be applicable to the general population. Therefore, further prospective cohort studies are still needed to clarify the causal association of endometriosis with asthma.

Although the exact pathophysiology for the relationship between endometriosis and asthma is unclear, existing studies have provide some plausible speculations for this association. There are abundant evidences that asthma is linked to Th2-type inflammatory responses induced by allergic stimuli, and the expression of interleukin (IL)-4 is crucial for the development of Th2 immune responses [26, 27]. The relationship of endometriosis with major Th2 cytokine immune responses has been reported in previous studies [28, 29]. In addition, asthma is related to the airways’ persistent inflammation, and for example, tumor necrosis factor-α, IL-4, transforming growth factor-β, IL-6, and vascular endothelial cell growth factor are all involved in the inflammatory response of asthmatic lungs as well as airway remodeling [27, 30, 31]. Inflammatory responses have also been observed to play important roles in the pathogenesis of endometriosis in several studies [15, 32], finding that women with endometriosis had higher levels of inflammatory cytokines in the peritoneum or serum than those without endometriosis [33, 34]. Both endometriosis and asthma were related to inflammatory and immunity response, indicating that women who had endometriosis may be potential high-risk population to develop asthma, and for them, asthma-related indicators should be focused on monitoring, and immunity should be improved to reduce the odds of asthma.

The subgroup analyses results showed the positive association of endometriosis with asthma was also observed in women aged 40–49 years old, with BMI of 25-29.9 kg/m^2^, and had pregnancy history. Age played an important role in adult asthma. In 2005–2018, in the U.S., the overall prevalence of asthma in youngers, adults, middle-aged adults, and elderly adults was 8.30%, 8.41%, 8.70%, and 7.92%, respectively [35]. For adult females, especially menopausal women aged 50 to 60 years old, there is a drop in asthma severity compared to men [36]. As sex hormones levels decrease with menopause, the age-adjusted risk of asthma may drop in postmenopausal compared to premenopausal women [37, 38]. According to our findings, peri-menopausal women who had endometriosis may be at higher risk of asthma. Tempest et al. [25] suggested the mean of BMI was little higher in the women with endometriosis than females without endometriosis. In a retrospective cohort study exploring the relationship of weight change patterns in adulthood with the incidence of asthma, overweight participants seemed to have a significantly higher risk of developing asthma than those had normal weight [39]. Similarly, we observed the association between endometriosis and asthma in women with BMI of 25-29.9 kg/m^2^ that is at the overweight status. In fact, compared with normal weight, both obesity and underweight had adverse effects on asthma control [40]. Although this relationship was not significant in BMI < 18.5 kg/m^2^ or ≥ 30 kg/m^2^ subgroup, it may be meaningful to recommended women who are underweight or obese to keep a healthy BMI through following healthy eating patterns and increasing physical activity levels suitably, and follow the routine physical examination, thereby reducing the potential risk of asthma [41]. Besides, no studies have discussed the association between endometriosis and asthma in women with or without a history of pregnancy. We speculated a possible mechanism that pregnancy may influence this association could be epigenetic regulation, as a previous study reported alterations in placental DNAm in women with antenatal asthma, compared with women without a history of asthma.

This study based on the NHANES database to explore the correlation between endometriosis and asthma, the study population is the relative representative population in the U.S. Also, we conducted subgroup analyses to further assesse the relationship between endometriosis and asthma in different age, race, BMI, and pregnancy history populations. However, there were some limitations in the current study. First, because of the observational nature of this research, it is hard to conclude a causal association of endometriosis with asthma. Second, due to the limitation of the database, both diagnoses of endometriosis and asthma were self-reported through questionnaires, and women without information on these two diseases were excluded, which may result in a selection bias. Third, this study only included the U.S. general population, further studies are needed to investigate the association of endometriosis with asthma in individuals with different races. In addition, information on endometriosis was only collected by the NHANES database in 1999–2006, and therefore, future prospective researches with larger samples and updated data are needed to explore the causal association of endometriosis with asthma.

## Conclusion

Women with endometriosis had potential risk of asthma. Monitoring asthma-related indicators in women who aged 40–49 years old, with BMI of 25-29.9 kg/m2, or having pregnancy history may be beneficial to reduce the odds of asthma. However, the causal association of endometriosis with asthma in adult women was needed to be further clarified.

## Electronic supplementary material

Below is the link to the electronic supplementary material.

Supplementary Material 1

## Data Availability

No datasets were generated or analysed during the current study.
