# Correction to: Association of endometriosis with asthma: A study of the NHANES database in 1999–2006

**DOI:** 10.1186/s41043-025-00798-2

**Published:** 2025-03-21

**Authors:** Guangxin Pan, Pei Zhang, Sha Li, Lanlan Cao, Changqun Yang

**Affiliations:** 1 Department of Obstetrics and Gynecology, The Central Hospital of Wuhan, Tongji Medical College, Huazhong University of Science and Technology, No 26. Shengli Street, Jiang’an District, Wuhan, 430014 Hubei Province P. R. China; 2 Key Laboratory for Molecular Diagnosis of Hubei Province, The Central Hospital of Wuhan, Tongji Medical College, Huazhong University of Science and Technology, Wuhan, 430014 P. R. China

**Correction to: Journal of Health, Population and Nutrition (2024)**

10.1186/s41043-024-00541-3

The original publication of this article [1] contained incorrect affiliations for the authors. The correct and incorrect affiliations are listed in this correction article.

Correct affiliations:

^1^Department of Obstetrics and Gynecology, The Central Hospital of Wuhan, Tongji Medical College, Huazhong University of Science and Technology, Wuhan 430014, P. R. China

^2^Key Laboratory for Molecular Diagnosis of Hubei Province, The Central Hospital of Wuhan, Tongji Medical College, Huazhong University of Science and Technology, Wuhan 430014, P. R. China

Incorrect affiliations:

^1^Department of Obstetrics and Gynecology, Tongji Medical College, The Central Hospital of Wuhan, Huazhong University of Science and Technology, No 26. Shengli Street, Jiang’an District, Wuhan 430014, Hubei Province, P. R. China

^2^Key Laboratory for Molecular Diagnosis of Hubei Province, Tongji Medical College, The Central Hospital of Wuhan, Huazhong University of Science and Technology, Wuhan 430014, P. R. China

## References

[1] Pan, G., Zhang, P., Li, S. *et al.* Association of endometriosis with asthma: a study of the NHANES database in 1999–2006. *J Health Popul Nutr* **43**, 50 (2024). 10.1186/s41043-024-00541-3 PMC11003178 38594768

