# Supplementary material for: Association of endometriosis with asthma: a study of the NHANES database in 1999–2006
Source: J Health Popul Nutr. 2024 Apr 9;43:50. doi: 10.1186/s41043-024-00541-3 (PMC11003178; doi:10.1186/s41043-024-00541-3)
Supplement: Supplementary file 1 — Supplementary Material 1 [file 41043_2024_541_MOESM1_ESM.docx]

**Supplementary table 1. The description of the missing data**

| Variable | Frequency | N (%) |
| --- | --- | --- |
| Education level | 5551 | 5 (0.09) |
| Marital status | 5404 | 152 (2.74) |
| PIR | 5195 | 361 (6.50) |
| Height | 5517 | 39 (0.70) |
| Weight | 5506 | 50 (0.90) |
| Waist circumference | 5457 | 99 (1.78) |
| BMI | 5498 | 58 (1.04) |
| Cigarette smoking | 5552 | 4 (0.07) |
| Sexual intercourse | 5447 | 109 (1.96) |
| Estrogen and progesterone hormones use | 5528 | 28 (0.50) |
| Uterine fibroids | 5538 | 18 (0.32) |
| Birth control pills use | 5552 | 4 (0.07) |

PIR: poverty income ratio, BMI: body mass index.
